# Dynamic Fracture and Fragmentation Characteristics of Metal Cylinder and Rings Subjected to Internal Explosive Loading

**DOI:** 10.3390/ma13030778

**Published:** 2020-02-08

**Authors:** Xuanyi An, Ping Ye, Jiayun Liu, Chao Tian, Shunshan Feng, Yongxiang Dong

**Affiliations:** State Key Laboratory of Explosion Science and Technology, Beijing Institute of Technology, Beijing 100081, China; 3120170152@bit.edu.cn (X.A.); 3120185185@bit.edu.cn (P.Y.); jiayun@bit.edu.cn (J.L.); 2120150235@bit.edu.cn (C.T.);

**Keywords:** fragmentation, microscopic analysis, copper plating, explosives, rupture strain

## Abstract

Dynamic fracture and fragmentation characteristics of explosively driven rings and cylinders are important issues in the field of weapon effectiveness and protection. However, the comparison of fracture characteristics between metal cylinder and rings, and the fracture characteristics of the metal shells at different axial positions, are rarely touched. In the present work, a recovery tank was used to collect fragments, and witness plates were used to investigate the fragment spatial distributions. Before the test, the representative positions of metal shells were plated with copper layers to locate the original position of the recovered fragments. After the test, scanning electron microscopy and optical microscope were used for characterizing the microstructure of the recovered fragments from different positions. Then, the recovered fragments were weighed and measured to investigate their mass and size characteristics. In addition, numerical simulation was used to further investigate the fracture mechanisms of explosively driven cylinders and rings. It was found that the projection angle axial distribution of the fragments for the metal cylinder was similar to that of the fragments for the metal rings. However, the fracture characteristics of the metal rings were significantly different from those of the metal cylinder. The adiabatic shear band played a key role in the fracture process of the metal cylinder, whereas the adiabatic shear band had little chance to initiate in the fracture process of the metal rings because the metal rings could deform uniformly with much fewer strain localizations due to their much lower length. The fracture surfaces of the fragments from different positions of the metal cylinder were very smooth, whereas dimples were found in the fracture surfaces of the fragments from different positions of the metal rings. The mass distribution of the fragments from the metal rings was more uniform than that of the fragments from the metal cylinder, and the circumferential rupture strains of the metal rings were much larger than those of the metal cylinder.

## 1. Introduction

The dynamic response process of expansion, fracture, and fragmentation of a metal shell subjected to internal explosive loadings is a highly complex phenomenon. After the detonation, the metal shell suffers strong shock loadings and large plastic deformations and eventually breaks into a large number of fragments with different masses, shapes, velocities, and projection angles. The fracture and fragmentation mechanisms of the metal shells under internal explosive loadings are associated with the characteristics of the fragments and thus directly influence the damage efficiency of fragmentation warheads. This dynamic subject has been of interest to researchers for decades, with lots of remarkable work.

Many models have been proposed to investigate the maximum fragment velocity of warheads [1,2,3,4], in which the Gurney formula [1] is the most widely used formula to calculate the maximum fragment velocity of warheads compactly filled with explosive charge. However, the Gurney formula is unsuitable for calculating the axial distribution of fragment velocities because the rarefaction waves from the ends of warhead can make the fragment velocities lower at the two ends. In the past decades, the axial distribution of fragment velocities has been extensively studied to determine the low velocities near the warhead edges [5,6,7,8,9,10], and Huang’s formula [7] showed high accuracy and wide applicability in the calculating of fragment velocity distribution along the axis of cylindrical casing under internal explosive loadings.

In terms of the fragmentation process of a metal shell subjected to internal explosive loadings, Mott [11] assumed that stress release waves will be generated on both sides of the fracture surface when the metal achieves the fracture strain. These release waves unload the metal surrounding the fracture, and the unloaded area cannot crack again. Beyond the unloaded area, the strain is still increasing, which leads to the probability of the fracture. After the entire metal shell is completely unloaded, new fractures will not be able to occur. Based on this assumption, Mott developed a model to calculate the size and the mass of the fragments. Grady [12,13] studied the statistical distribution of the fragments based on the energy conservation method and proposed a relationship between the strain rate of blast loading and the fragmentation scale. Taylor [14] proposed a model of fracture strain. In this model, the fractures initiate at the outer surface of the casing where the hoop stresses are tensile and then propagate inwardly, and penetrate through the casing when the stresses at the inner surface are converted from compressive to tensile. Hoggatt [15] investigated the fracture behavior of warheads and found that the shear fracture is the main fracture mode at high strain rate, which has been confirmed by subsequent researchers [16,17,18]. Tang [19] experimentally investigated the effects of the strain rate (changed from 2.5 × 10^4^ s^−1^ to 8.4 × 10^4^ s^−1^) on the fracture mechanisms of metal shells subjected to internal explosive loadings and found that as the strain rate increases, the fracture mode is converted from purely tensile to tensile–shear mixed and then to purely shear fracture. Recently, Liu [20] investigated the fracture mechanisms of explosively driven cylinder (under the strain rate of ~5 × 10^4^ s^−1^) by experiments and numerical simulation and found that the adiabatic shear band plays a key role in its fracture process. After the detonation, the adiabatic shear bands firstly initiate at the inner surface of the casing and then propagate outwardly along the maximum shear stress direction. Finally, the cracks initiate near the outer surface of the cylinder and propagate inwardly along the developed adiabatic shear bands. In addition, for investigating the dynamic behavior of the metal shells under high strain rate, there are other methods besides the explosive loading. For example, Avriel [21] conducted magnetically driven expanding cylinder experiments and measured the strength of materials at very high strain rates.

The fracture process of metal shell subjected to internal explosive loadings is very rapid and complex, and the fracture mechanisms of the metal shell have been extensively investigated as mentioned above. However, in the existing literature, fragments were all randomly chosen to study their fracture mechanisms, which cannot enable us to know the original position of the fragments. Due to the influence of rarefaction waves from the ends of warhead, the metal at different positions along the axis of warhead suffers different loadings and thus may present different fracture mechanisms. In addition, the comparison of fracture mechanisms between metal cylinder and rings is also rarely touched. The fracture mechanisms of the metal rings and cylinder at different positions are critical for the design of munitions and armaments. Therefore, it is essential to locate the original position of the recovered fragments and investigate the fracture characteristics of the fragments from different positions and further compare the fracture mechanisms between metal cylinder and rings. In the present work, the representative positions of metal shells were plated with copper layers with different widths to locate the original position of the recovered fragments. Then, we investigated the dynamic fracture and fragmentation characteristics of explosively driven cylinder and rings by using a recovery tank, witness plates, scanning electron microscopy (SEM), and optical microscope. In addition, numerical simulation was used to further investigate the fracture mechanisms of explosively driven cylinders and rings.

## 2. Experimental Design

**Figure 1:**
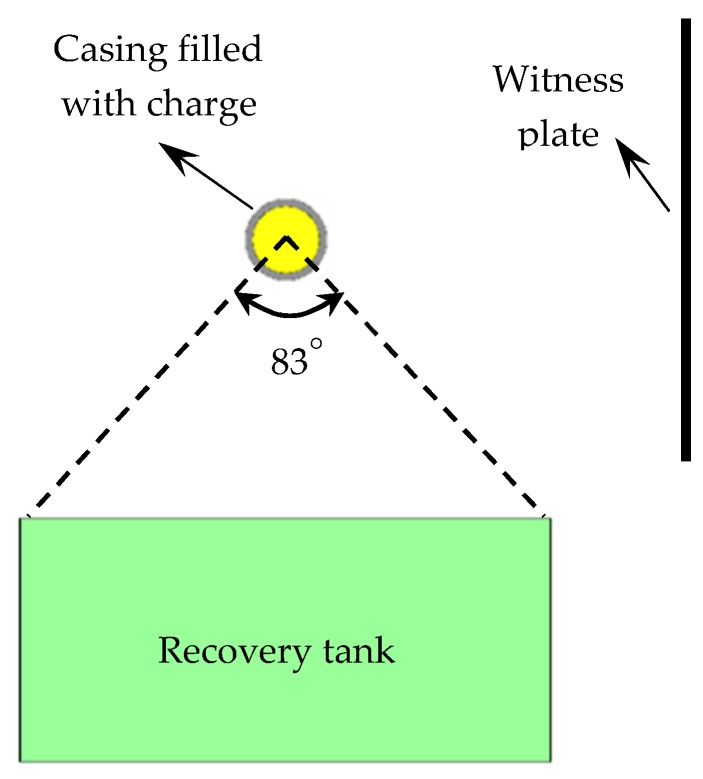
Schematic of experimental setup.

To investigate the dynamic fracture and fragmentation characteristics of explosively driven cylinder and rings, experiments were conducted on an explosion setup as shown in Figure 1. The fragment spatial distribution was determined with the holes in witness plate pierced by the fragment impact. The witness plate was made of carbon structural steel (235A steel, density 7.85 g/cm^3^, elastic modulus 210 GPa, Poisson’s ratio 0.3, yield strength 235 MPa) with the size of 1100 mm long, 1100 mm high, and 2 mm thick. The recovery tank filled with water was positioned below the specimen and collected a fraction of metal fragments. Then, the mass recovery rate could be calculated from Equation (1). After the test, the recovered fragments were weighed and measured to investigate their mass and size characteristics, and scanning electron microscopy (SEM) and optical microscope were used for characterizing their fracture surfaces and microstructure.

(1) $$\eta =m_{R}/(m\cdot \frac{83^{{}^{\circ}}}{360^{{}^{\circ}}})$$

where *η* is the mass recovery rate, *m_R_* is the total mass of recovered fragments, and *m* is the total mass of metal shell.

**Figure 2:**
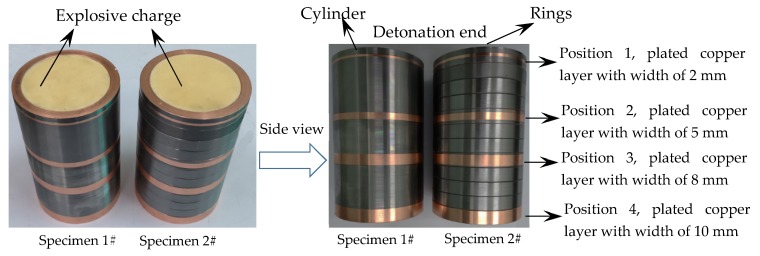
Details of the specimens.

An axisymmetric cylindrical geometry was used in the present work as shown in Figure 2. Before the test, the representative positions of metal shells were plated with copper layers with different widths as shown in Figure 2. The thickness of the plated copper layers was about 0.03 mm, so the influences of the copper plating on the mechanical properties of metal shells could be neglected. After the test, fragments with the plated copper layers could be found, and thus we could locate the original position of the recovered fragments. The explosive charge, which had a diameter of 60 mm and a length of 120 mm, was Composition B (COMP-B) (1.717 g/cm^3^, detonation pressure 29 GPa, detonation velocity 7980 m/s). The metal shells with two different lengths were designed as 60 mm in internal diameter and 6.5 mm in thickness. The cylinder length of Specimen 1# was 120 mm, and Specimen 2# consisted of 12 rings with lengths of 10 mm. 40 Cr steel (as received) was used as the metal shells, and its material properties are presented in Table 1. In the experiments, the warhead was initiated at the center of one end. For the explosively driven metal shells, the strain rate can be calculated as *V*/*r* [22], where *V* is the initial fragment velocity, and *r* is the initial radius of the metal shell. Therefore, the expansion strain rate of the metal shell in the present work is ~5.2 × 10^4^ s^−1^ after detonation of the explosive charge.

## 3. Experimental Results and Discussion

### 3.1. Witness Plate

**Figure 3:**
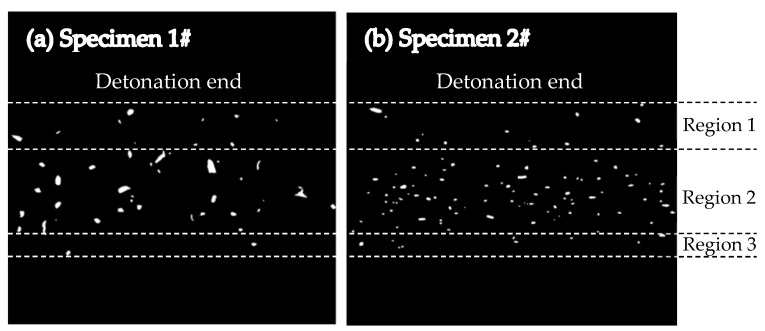
Witness plates showing hole distributions: (**a**) Witness plate showing hole distribution for Specimen 1#; (**b**) Witness plate showing hole distribution for Specimen 2#.

The fragment spatial distribution was determined with the holes in witness plate pierced by the fragment impact. In order to get reliable data, the distortion and deformation of witness plates were removed manually. Then the front surfaces of the plates were painted black and a photograph was taken with illumination from behind each plate. And finally, the images obtained in this way were converted into grayscale bitmaps (Figure 3), and the conversion was carried out in accordance with the methodology described in [10], using Arnold’s method [23]. As shown in Figure 3, the number of holes pierced by the fragments from Specimen 2# was larger than that of holes pierced by the fragments from Specimen 1#, and the hole size distribution for Specimen 2# was more uniform. The distributions of the holes impacted by fragments of the specimens could be divided into three regions, i.e., Region 1, Region 2, and Region 3. The holes in Region 1 and Region 3 were pierced by the fragments from the detonation end of specimens and those from the non-detonation end of specimens, respectively, and the holes in Region 2 were pierced by the fragments from the middle part of specimens. The holes in Region 1 and Region 3 were more scattered than those in Region 2 because the projection angles of fragments from the ends were bigger than those of fragments from the middle part [24,25]. It can be seen that the projection angle axial distribution of the fragments for the metal cylinder was similar to that of the fragments for the metal rings, inferred from the similar site distributions of the holes in witness plates (Figure 3).

### 3.2. Fracture Mechanism

**Figure 4:**
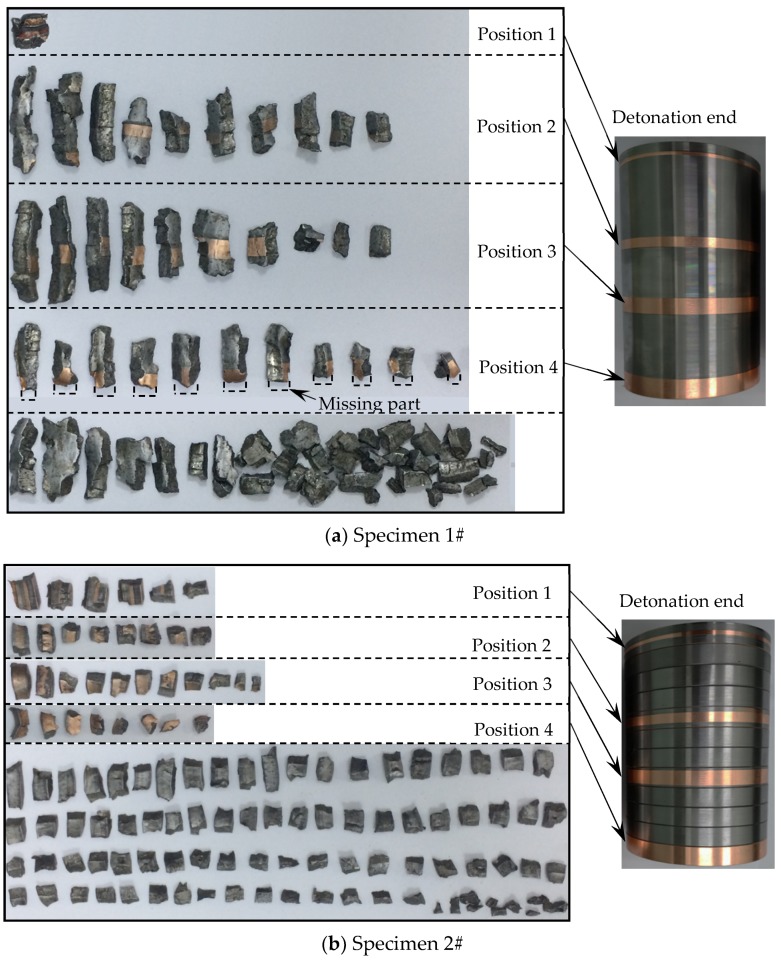
Recovered fragments and their original positions: (**a**) Recovered fragments and their original positions for Specimen 1#; (**b**) Recovered fragments and their original positions for Specimen 2#.

After the explosion test, the recovered fragments from the recovery tank were cleaned, dried, and counted. Seventy-two fragments and 121 fragments were recovered for Specimen 1# and Specimen 2#, respectively, and the mass recovery rates (Equation (1)) were 65.1% and 79.7% for Specimen 1# and Specimen 2#, respectively. The plated copper layers with different widths could be easily distinguished, and thus the original position of the recovered fragments with the plated copper layers could be known as shown in Figure 4. It can be seen that the aspect ratio of the recovered fragments for Specimen 1# ranged from 1 to 7, whereas that of the recovered fragments for Specimen 2# had a small range (just from 1 to 2.5). The axial length was bigger than the circumferential width for most fragments of the metal cylinder (Specimen 1#), whereas the circumferential width was bigger than the axial length for the most fragments of the metal rings (Specimen 2#).

**Figure 5:**
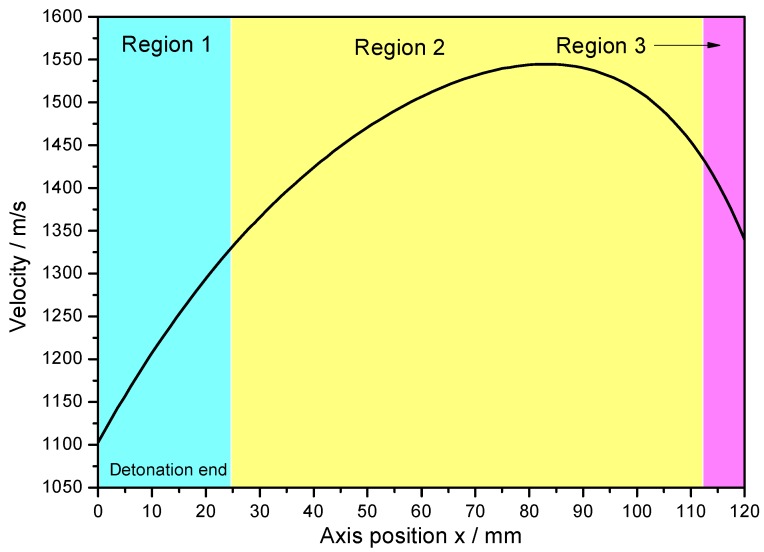
Fragment velocity axial distribution.

**Figure 6:**
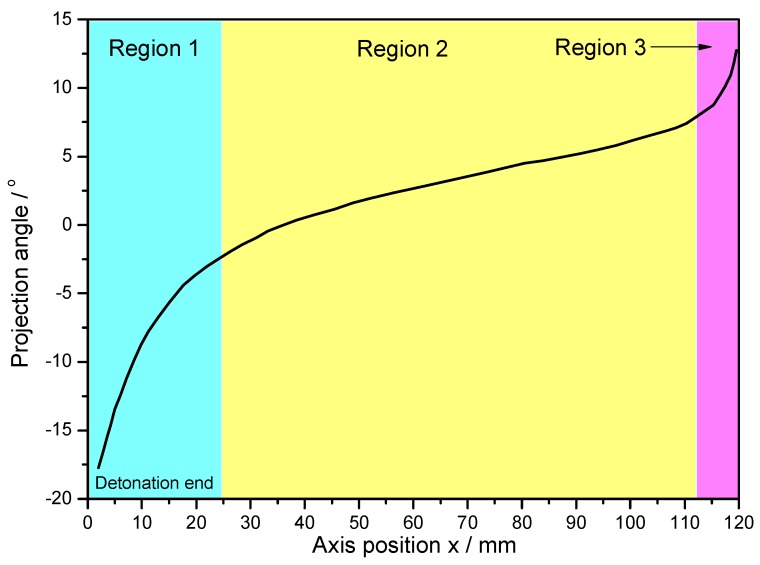
Projection angle axial distribution.

For the metal cylinder, due to the radical changes of both the fragment velocity (calculated by Hung’s formula [7] as shown in Equation (2)) and the fragment projection angle (calculated by König’s formula [24] as shown in Equation (3)) at the two ends (the Region 1 and Region 3 as shown in Figure 5 and Figure 6) caused by the effects of the rarefaction waves, the large shear deformation and tensile deformation occurred in Region 1 and Region 3, resulting in relatively small fragments at the two ends (Figure 3 and Figure 4). Although the fragment velocity varied a lot in the middle part of the warhead (Region 2) (Figure 5), most of the fragments from the middle part of the warhead were long and slender because of the little changes of the projection angle in the middle part of the warhead (Region 2) as shown in Figure 6. In contrast, the axial lengths of the fragments for the metal rings were similar because the metal rings were disconnected in the axial direction of the warhead. The variation range of projection angle at the detonation end was larger than that at the non-detonation end (Figure 6), and thus the fragments from the detonation end were more scattered than those from the non-detonation end (Figure 3).

(2) $$V_{Huang}=(1-0.361e^{-1.111x/d})\cdot (1-0.192e^{-3.03(L-x)/d})\cdot \sqrt{2E}\cdot \sqrt{\frac{1}{1/\beta +0.5}}$$

where *V_Huang_* is the initial fragment velocity at a certain point on the cylindrical casing, *x* is the distance between the point and the detonation end along cylinder axis, *L* is the length of casing, *d* is the diameter of charge, $\sqrt{2E}$ is the Gurney constant of explosive charge, and *β* is the mass ratio of explosive charge to metallic casing.

(3) $$\left\{ \begin{array}{l} \sin^{\alpha }=(V/(2D))\cdot \cos^{i}\\ \alpha_{c}=-2.5\cdot (\left|d(F_{x})/dx\right|{)}^{m}\cdot (k_{1}\cdot {\left(1/\beta +1/2\right)}^{1/2})\\ \alpha_{c}{}^{\prime }=+2.5\cdot (\left|d(F_{x})/dx\right|{)}^{m}\cdot (k_{2}\cdot {\left(1/\beta +1/2\right)}^{1/2})\\ F_{x}=(1-{\left(1-n_{1}\cdot {\left(x/d\right)}^{n_{1}}\right)}^{2})\cdot (1-{\left(1-2n_{2}\cdot {\left((L-x)/d\right)}^{n_{2}}\right)}^{2}) \end{array}\right.$$

where α is Taylor angle [26], *V* is the fragment velocity, *D* is the detonation velocity of the explosive, *i* is the angle between the detonation front and the normal to the explosive/metal interface, $\alpha_{c}$ and $\alpha_{c}{}^{\prime }$ are correction terms for the Taylor angle at the detonation end and the non-detonation end respectively, *m* is the parameter about the type of the casing, and *k*_1_, *k*_2_, *n*_1_, and *n*_2_ are coefficients.

**Figure 7:**
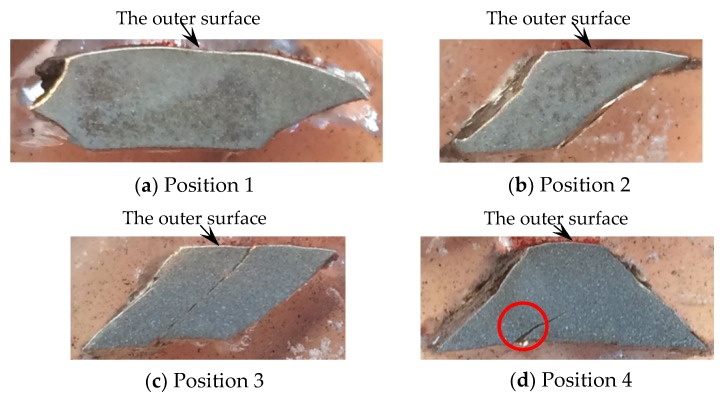
Cross-sections of typical fragments from different positions of Specimen 1#: (**a**) Cross-section of typical fragment from position 1; (**b**) Cross-section of typical fragment from position 2; (**c**) Cross-section of typical fragment from position 3; (**d**) Cross-section of typical fragment from position 4.

**Figure 8:**
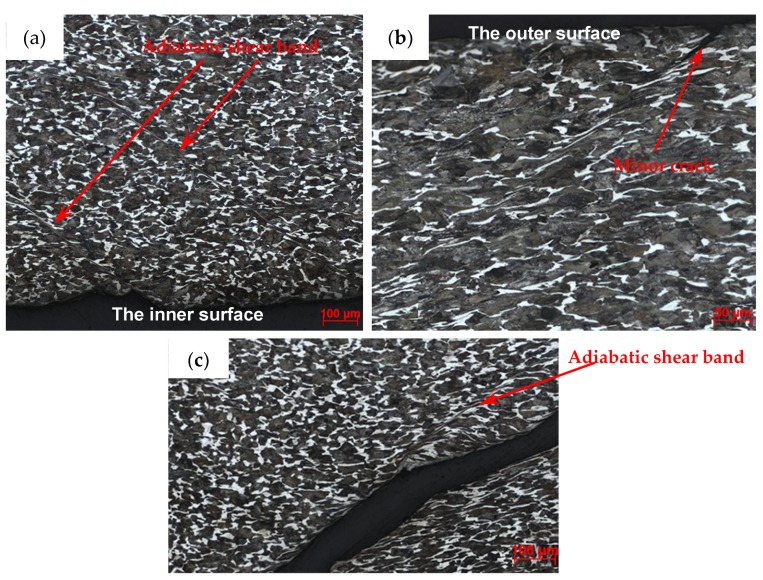
Typical results of optical microscope for Specimen 1#. (**a**) Multiple adiabatic shear bands near the inner surface; (**b**) minor crack near the outer surface; (**c**) adiabatic shear band within the crack, which is the magnified region of the red circle in Figure 7d.

**Figure 9:**
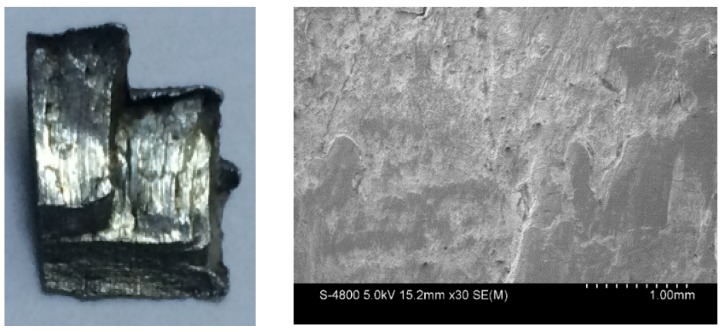
Typical fracture surface of fragment from Specimen 1# (left) and its morphology captured by SEM (right).

For the metal cylinder (Specimen 1#), typical fragments from different positions were sectioned, ground, and polished. The cross-sections, which are perpendicular to the axis of the casing, of the fragments from different positions, are shown in Figure 7. It can be clearly seen that the fractures were typically oriented at ~45° with respect to either the inner or outer fragment surfaces. Some cracks were observed within some fragments as shown in Figure 7c,d. The crack within the fragment from Position 3 (Figure 7c) propagated the entire way through the material (i.e., it spanned the fragment thickness), whereas the crack within the fragment from Position 4 (Figure 7d) did not span the fragment thickness. In order to obtain more reliable results, different fragments were tested by optical microscope for each position of Specimen 1#. For the fragments from different positions of Specimen 1#, the results of optical microscope were similar. The typical results of optical microscope are shown in Figure 8. Multiple adiabatic shear bands, which were oriented at ~45° with respect to the inner surface, were observed as shown in Figure 8a. These adiabatic shear bands were located near the inner surface of the fragment and did not span the fragment thickness. Therefore, it can be concluded that these adiabatic shear bands initiated near the inner surface and propagated towards the outer surface. Inside the adiabatic shear bands, the grains were strongly deformed, whereas the grains outside the adiabatic shear bands were nearly the same with their original states. It can be seen that the inner surface of the fragment was step-like, which shows that the adiabatic shear band could maintain a long time before it converged into a crack, and the material at two sides of the adiabatic shear band moved a long distance along the adiabatic shear band during this time. Near the outer surface, some minor cracks were observed as shown in Figure 8b. Within the big crack marked with a red circle in Figure 7d, adiabatic shear band was also observed as shown in Figure 8c. Scanning electron microscopy (SEM) was used to examine the morphologies of the fracture surfaces. In order to obtain more reliable results, different fragments were tested by SEM for each position of Specimen 1#. The results showed that the morphologies of the fracture surfaces of fragments from different positions of Specimen 1# were similar, and the fracture surfaces were very smooth, which was caused by the adiabatic shear bands. The typical fracture surface of fragment and its morphology are shown in Figure 9. Therefore, the process of fragmentation of the metal cylinder can be described as follows: after the detonation, the cylinder moved outward rapidly and deformed at a high strain rate. The adiabatic shear band played a key role in the fracture process of the metal cylinder, and it preferred to initiate near the inner surface and propagated towards the outer surface along the maximum shear stress direction. During the expansion of the cylinder, the adiabatic shear bands could maintain a long time, and the material at two sides of the adiabatic shear bands moved a long distance along the adiabatic shear bands during this time. As the further expansion of the cylinder occurred, the plastic deformation exceeded a certain value because of the slippage of the material along the adiabatic shear band, and then some adiabatic shear bands converged into cracks. At the same time, some cracks initiated near the outer surface because of the tensile stress and propagated towards the inner surface. Then, stress release waves were generated on both sides of the cracks. These release waves unloaded the metal surrounding the fracture so that the unloaded area could not crack again. There still were some adiabatic shear bands, which did not converge into cracks and are the ones observed in the fragments (Figure 8), within the unloaded area. After the cracks propagated through the thickness of the cylinder, the cylinder was completely broken.

**Figure 10:**
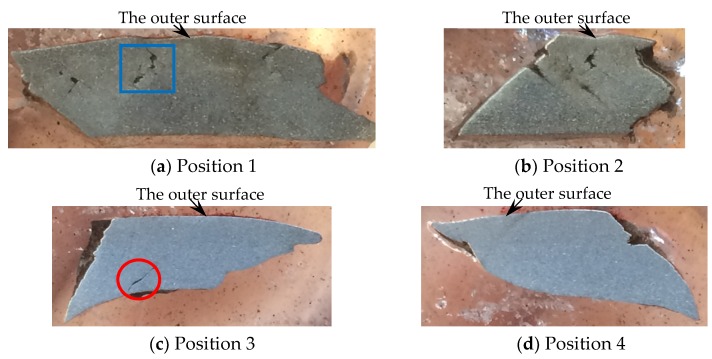
Cross-sections of typical fragments from different positions of Specimen 2#: (**a**) Cross-section of typical fragment from position 1; (**b**) Cross-section of typical fragment from position 2; (**c**) Cross-section of typical fragment from position 3; (**d**) Cross-section of typical fragment from position 4.

**Figure 11:**
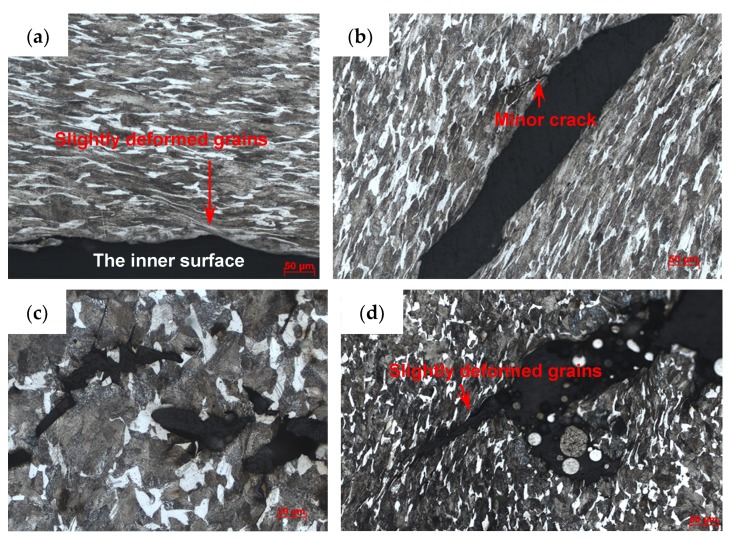
Typical results of optical microscope for Specimen 2#. (**a**) Slightly deformed grains near the inner surface; (**b**) minor crack within the big crack, which is the magnified region of the red circle in Figure 10c; (**c**) internal brittle microvoid damage, which is the magnified region of the blue square in Figure 10a; (**d**) internal ductile microvoid damage.

**Figure 12:**
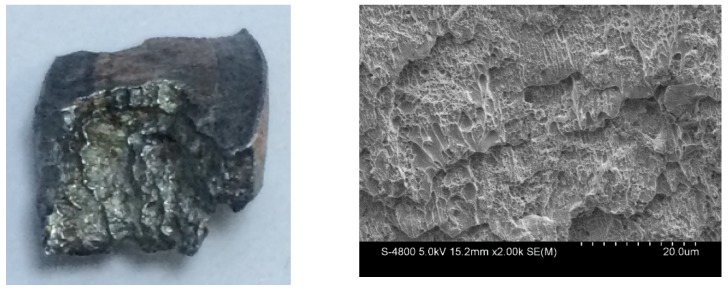
Typical fracture surface of fragment from Specimen 2# (left) and its morphology captured by SEM (right).

For the metal rings (Specimen 2#), the deformation and fracture characteristics were significantly different from those of the metal cylinder. The cross-sections, which are perpendicular to the axis of the casing, of the fragments from different positions, are shown in Figure 10. Some fractures were typically oriented at ~45° with respect to either the inner or outer fragment surfaces, and some fractures were perpendicular to the outer surface. In order to obtain more reliable results, different fragments were tested by optical microscope for each position of Specimen 2#. For the fragments from different positions of Specimen 2#, no adiabatic shear band was observed, but some slightly deformed grains, which made the inner surface of the fragment step-like, were observed near the inner surface as shown in Figure 11a. Within a big fracture which did not span the fragment thickness as marked with the red circle in Figure 10c, a minor crack was observed and no adiabatic shear band was found (Figure 11b). Large and small voids were observed within the fragments from Position 1 and Position 2 as shown in Figure 10a,b. Some voids were parallel to the radial direction, and some voids were at an angle of ~45° to the radial direction. The grains around some voids were nearly the same with their original states (Figure 11c), which shows that these voids were caused by brittle fracture. The grains around other voids were slightly deformed (Figure 11d), which shows that these voids were caused by ductile fracture. In order to obtain more reliable results, different fragments were tested by SEM for each position of Specimen 2#. The morphologies of the fracture surfaces of fragments from different positions of Specimen 2# were similar, and dimples were found in the fracture surfaces, which shows that it was a kind of ductile fracture. The typical fracture surface of fragment and its morphology are shown in Figure 12. Therefore, the process of fragmentation of the metal rings can be described as follows: after the detonation, the rings moved outward and deformed at a high strain rate. During the expansion of the metal rings, the adiabatic shear band had little chance to initiate, but some slightly deformed grains formed near the inner surface. As the further expansion of the metal rings, the cracks formed when the plastic deformation exceeded a certain value. At the same time, large and small voids initiated within some fragments by brittle fracture or ductile fracture. After the cracks propagated through the thickness of the rings, the metal rings were completely broken.

**Figure 13:**
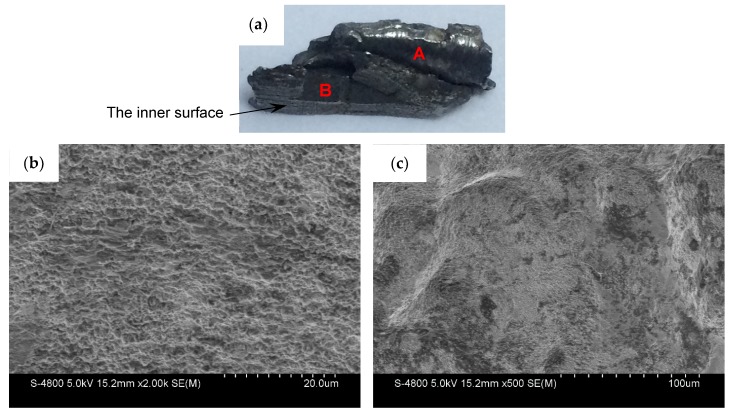
(**a**) Axial fracture surface of fragment from the detonation end of Specimen 1#; (**b**) metallurgical characterization of Site A in (**a**); (**c**) metallurgical characterization of Site B in (**a**).

**Figure 14:**
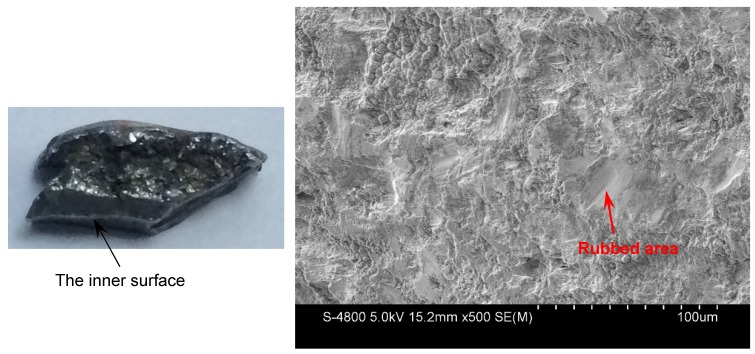
Axial fracture surface of fragment from the non-detonation end of Specimen 2# (left) and its morphology captured by SEM (right).

The fragments from the detonation ends (Position 1) of both Specimens 1# and 2# deformed significantly due to the effects of the detonation products leaking out from the free surfaces at the detonation ends, and the fragment from the detonation end of Specimen 1# was split along the thickness of the casing as shown in Figure 13a. The morphologies of different sites (A and B) are shown in Figure 13b,c. It can be clearly seen that it was a kind of ductile fracture, and some of the material was twisted due to the complex stress state (Figure 13c). The fragments from the non-detonation end (Position 4) of Specimen 1# were very small, but unfortunately, they were lost, as shown in Figure 4a. The metal ring at the non-detonation end (Position 4) of Specimen 2# was split along the axis of the warhead as shown in Figure 4b. This phenomenon is due to the radical changes of both the fragment velocity and the fragment projection angle (Region 3 as shown in Figure 5 and Figure 6). Typical axial fracture surface of fragment from the non-detonation end of Specimen 2# and its morphology captured by SEM are shown in Figure 14. Some areas were flat due to the rub by its adjacent fragment (Figure 14).

### 3.3. Fragment Mass and Size

**Figure 15:**
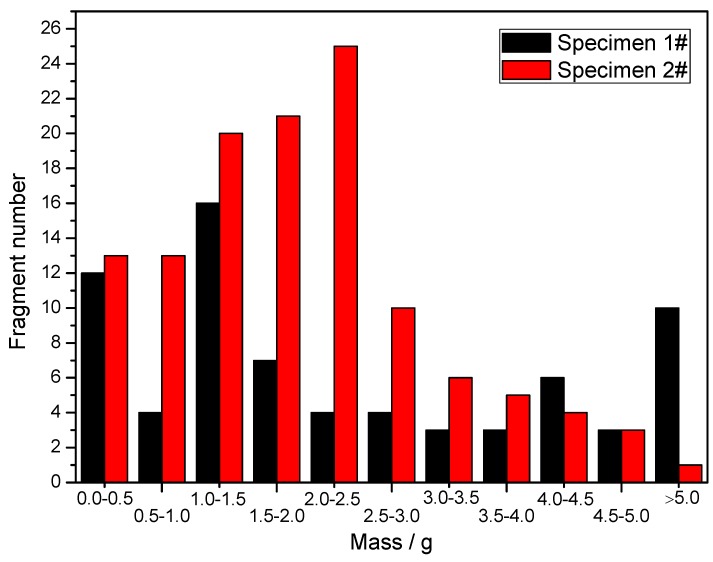
Fragment mass distribution of specimens.

After the explosion test, all of the recovered fragments were weighed to investigate their mass distributions, and the fragments (with both inner and outer surfaces) from different positions were measured to investigate their size characteristics. The fragment mass distributions are shown in Figure 15. It can be clearly seen that the majority of the recovered fragments from Specimen 2# were under 3 g, and the number of fragments with big masses was very small. In contrast, for Specimen 1#, the recovered fragments larger than 3 g accounted for a large proportion of the total number of recovered fragments. These results indicate that the mass distribution of the fragments from the metal rings was more uniform than that of the fragments from the metal cylinder.

The size characteristics of the recovered fragments from different positions are shown in Table 2. It can be clearly seen that the average thicknesses of fragments from different positions of both Specimen 1# and Specimen 2# were at the same level. The average thicknesses of the fragments from the ends (Positions 1 and 4) of specimens were slightly larger than those from the middle part (Positions 2 and 3) of the specimens because the rarefaction waves from the ends weakened the driving capability of explosion products at the ends, and thus the compressive stresses on the shells were lower at the ends. The average widths of the plated copper layers for the middle part (Positions 2 and 3) of specimens are shown in Table 2, but unfortunately, those for the ends (Positions 1 and 4) of specimens were not obtained because the deformation of fragments from the ends was too large to measure the average widths of the plated copper layers. The average widths of the plated copper layers for Specimen 1# were slightly larger than their original ones because tensile stress existed between the fragments along the axis of the cylinder during its expansion due to the changes of both the fragment velocity and the fragment projection angle along the axis of the cylinder. In contrast, the average widths of the plated copper layers for Specimen 2# were smaller than their original ones, because the metal rings were disconnected in the axial direction of the warhead. The casings reached their maximum expansion radii when they were completely broken. Assuming that the density of the metal was a constant during the expansions of the metal shells, and the fragments could maintain their size characteristics after the metal shells were completely broken. Therefore, the circumferential rupture strains of the casings can be calculated according to the average thicknesses of the fragments and the average widths of the plated copper layers as shown in Table 2. Then the radius at rupture could be further calculated, and the ratios of rupture radius to original radius for different positions of specimens are shown in Table 2. It can be clearly seen that the circumferential rupture strains for different positions of the metal rings were at the same level (~78%), and those of the metal cylinder were much lower (~41%). The metal cylinder was broken when it expanded to ~1.41 times its original radius, and the metal rings were broken when they expanded to ~1.78 times their original radius.

## 4. Numerical Simulation

In order to understand the reason why the deformation and fracture characteristics of metal rings were significantly different from those of the metal cylinder, the finite difference engineering package AUTODYN (14.0) was used to further investigate the fracture mechanisms of explosively driven cylinders and rings. It has been reported that eight-node solid elements can be used to simulate the dynamic behavior of the metal shells under internal explosive loading [8], and smoothed particle hydrodynamics (SPH) can be used to simulate the explosive charge [8,10]. Therefore, in the present work, the metal shells were modeled with eight-node solid elements, and the explosive charge was modeled with smoothed particle hydrodynamics (SPH). The element size and the particle size in the present work were set as 0.3 mm, which is small enough to produce accurate results [4,10]. In the present work, a quarter model of each specimen was established due to the symmetry of the specimens. The 40 Cr steel was simulated by using the Johnson–Cook model [27], and the material parameters of the 40 Cr steel in our numerical simulation were adopted from reference [28]. The equation of state (EOS) of explosive charge was described with the “JWL” model (a function of relative volume and internal energy per initial volume), and the material parameters of the explosive charge in our numerical simulation were adopted from reference [29].

**Figure 16:**
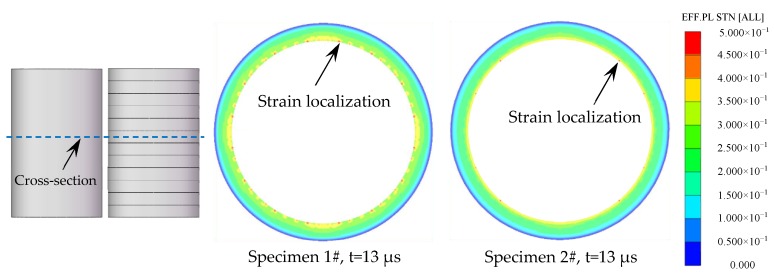
The effective plastic strain of the representative cross-section of the specimens.

The effective plastic strain of representative cross-sections, which are perpendicular to the axis of the Specimens 1# and 2#, are shown in Figure 16. It can be clearly seen that, after the detonation waves arrived at the cross-sections, many strain localizations formed at the inner surface of Specimen 1#, whereas the inner surface of Specimen 2# had much fewer strain localizations. It has been reported that the strain localizations can cause the probability of the fracture [30,31]. Therefore, it can be concluded that it was easier to form strain localizations at the inner surface of the metal cylinder (Specimen 1#), resulting in multiple adiabatic shear bands located near the inner surface (Figure 8a). However, for the metal rings (Specimen 2#), it was easier to deform uniformly with much fewer strain localizations because the length of the rings was much lower, and thus the adiabatic shear band had little chance to initiate, but some slightly deformed grains formed at the inner surface (Figure 11a). As a result of the fact that the metal rings (Specimen 2#) could deform uniformly with much fewer chances of formation of the strain localizations and the adiabatic shear bands, the metal rings ruptured at larger radii and had larger circumferential rupture strains compared to the metal cylinder (Specimen 1#) as shown in Table 2.

**Figure 17:**
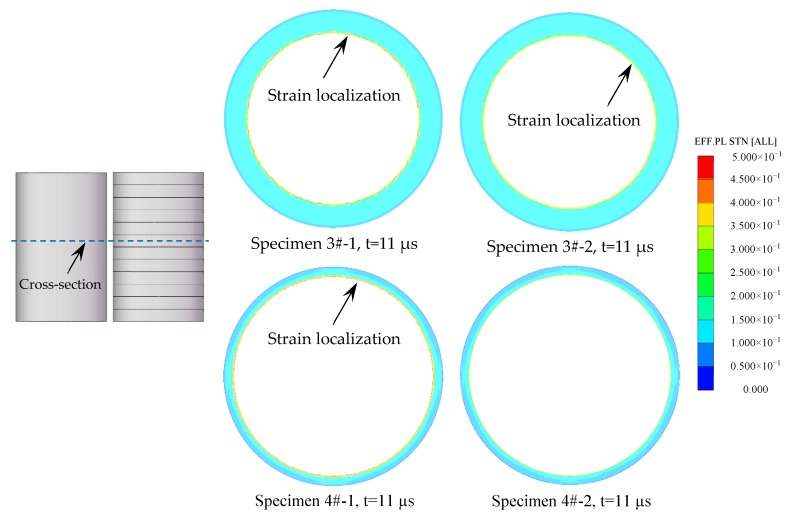
The effective plastic strain of the representative cross-section of the specimens.

In order to demonstrate the reproducibility of the results in the present work, additional two sets of specimens were tested by numerical simulation as shown in Table 3, where the Specimen 3#-1 and Specimen 4#-1 were metal cylinders, and Specimen 3#-2 and Specimen 4#-2 consisted of 12 rings with lengths of 10 mm each. The effective plastic strain of representative cross-sections, which are perpendicular to the axis of the specimens, are shown in Figure 17. It can be seen that, after the detonation waves arrived the cross-sections, many strain localizations formed at the inner surface of the metal cylinders (Specimen 3#-1 and Specimen 4#-1), whereas the inner surface of the metal rings (Specimen 3#-2 and Specimen 4#-2) deformed uniformly with much fewer strain localizations. These results were consistent with those of Specimens 1# and 2#.

## 5. Conclusions

In the present work, the dynamic fracture and fragmentation characteristics of explosively driven cylinder and rings were investigated experimentally and numerically. The representative positions of metal shells were plated with copper layers with different widths to locate the original position of the recovered fragments. A recovery tank filled with water was used to collect fragments, and witness plates were used to investigate the fragment spatial distributions. Then, SEM and optical microscope were used to investigate the fracture mechanisms of the metal shells at different positions, and the recovered fragments were weighed and measured to investigate their mass and size characteristics. In addition, numerical simulation was used to further investigate the fracture mechanisms of explosively driven cylinders and rings. The following conclusions can be drawn from current study.

(1) The similar site distributions of the holes in witness plates suggest that the projection angle axial distribution of the fragments for the metal cylinder was similar to that of the fragments for the metal rings. The fragments from the two ends of the metal cylinder were relatively small, and the metal ring at the non-detonation end of the metal rings was split along the axis of the warhead, which is due to the radical changes of both the fragment velocity and the fragment projection angle along the axis of the casings at the ends.
(2) For the metal cylinder, the fracture surfaces were typically oriented at ~45° with respect to either the inner or outer fragment surfaces. The adiabatic shear band played a key role in the fracture process of the metal cylinder, and it preferred to initiate near the inner surface and propagated towards the outer surface along the maximum shear stress direction. The adiabatic shear band could maintain a long time before it converged into a crack, and the material at two sides of the adiabatic shear band moved a long distance along the adiabatic shear band during this time, which made the inner surface of the fragment step-like. The morphologies of the fracture surfaces of fragments from different positions of metal cylinder were similar, and the fracture surfaces were very smooth, which was caused by the adiabatic shear bands.
(3) For the metal rings, the deformation and fracture characteristics were significantly different from those of the metal cylinder. Some fractures were typically oriented at ~45° with respect to either the inner or outer fragment surfaces, and some fractures were perpendicular to the outer surface. Large and small voids formed within some fragments by brittle fracture or ductile fracture. During the expansion of the metal rings, the adiabatic shear band had little chance to initiate because the metal rings could deform uniformly with much fewer strain localizations due to their much lower length, but some slightly deformed grains formed near the inner surface, which made the inner surface of the fragment step-like. The morphologies of the fracture surfaces of fragments from different positions of the metal rings were similar, and dimples were found in the fracture surfaces, which shows that it was a kind of ductile fracture.
(4) The mass distribution of the fragments from the metal rings was more uniform than that of the fragments from the metal cylinder. The average thicknesses of the fragments from different positions of the metal cylinder and rings were at the same level. The average widths of the plated copper layers for the metal cylinder were slightly larger than their original ones because tensile stress existed between the fragments along the axis of the cylinder during its expansion. In contrast, the average widths of the plated copper layers for the metal rings were smaller than their original ones, because the metal rings were disconnected in the axial direction of the warhead.
(5) Under current experimental conditions, the circumferential rupture strains for different positions of the metal rings were at the same level (~78%), and those of the metal cylinder were much lower (~41%). The metal cylinder was broken when it expanded to ~1.41 times its original radius, and the metal rings were broken when they expanded to ~1.78 times their original radius.

The dynamic fracture and fragmentation characteristics of explosively driven metal shells were investigated under current experimental and numerical conditions. The fracture mechanisms of the explosively loaded shells are very complex because of the randomness and uncertainty of the fracture point and the fracture path. Therefore, more experiments and numerical simulation are expected to be conducted for more accurate and reliable results, and thus will be investigated in our further studies based on the experimental and numerical results presented in the present work. In addition, more experimental devices (such as high-speed camera) and alternative methods (such as electromagnetic expanding cylinders) will be tried in our subsequent research.

## Figures and Tables

**Table 1:** Material properties of 40 Cr steel.

Density (g/cm^3^)	Yield Strength (MPa)	Elastic Modulus (GPa)	Hardness	Poisson Ratio
7.87	785	211	207 HB	0.3

**Table 2:** Size characteristics of the recovered fragments from different positions.

No.	Position	Average Thickness (mm)	Average Width of the Plated Copper Layers (mm)	Circumferential Rupture Strain (%)	Ratio of Rupture Radius to Original Radius
1#	1	4.71	-----	-----	-----
	2	4.59	5.07	39.7	1.397
	3	4.48	8.14	42.7	1.427
	4	4.64	-----	-----	-----
2#	1	4.82	-----	-----	-----
	2	4.47	4.12	76.4	1.764
	3	4.48	6.43	80.5	1.805
	4	5.05	-----	-----	-----

**Table 3:** Parameters of the additional two sets of specimens.

No.	Length of Charge (mm)	Length of Rings (mm)	Diameter of Charge (mm)	Outside Diameter of Casing (mm)
3#-1	120	120	60	80
3#-2	120	10	60	80
4#-1	120	120	60	66
4#-2	120	10	60	66

## References

[1] Gurney R.W. (1943). The Initial Velocities of Fragments from Bombs, Shells and Grenades.

[2] Zhang Q., Miao C.Q., Lin D.C., Bai C.H. (2003). Relation of fragment with air shock wave intensity for explosion in a shell. Int. J. Impact Eng..

[3] Elek P., Jaramaz S., Mickovic D. (2013). Modeling of the metal cylinder acceleration under explosive loading. Sci. Tech. Rev..

[4] An X.Y., Dong Y.X., Liu J.Y., Tian C. (2018). General formula to calculate the fragment velocity of warheads with hollow core. Int. J. Impact Eng..

[5] Hennequin E. Influence of edge effects on the initial velocities of fragments from a warhead. Proceedings of the 9th international symposium on ballistics.

[6] Charron Y.J. (1979). Estimation of velocity distribution of fragmenting warheads using a modified Gurney method. Master’s Thesis.

[7] Huang G.Y., Li W., Feng S.S. (2015). Axial distribution of fragment velocities from cylindrical casing under explosive loading. Int. J. Impact Eng..

[8] Grisaro H., Dancygier A.N. (2015). Numerical study of velocity distribution of fragments caused by explosion of a cylindrical cased charge. Int. J. Impact Eng..

[9] Ning J.G., Duan Y., Xu X., Ren H.L. (2017). Velocity characteristics of fragments from prismatic casing under internal explosive loading. Int. J. Impact Eng..

[10] An X.Y., Liu J.Y., Ye P., Tian C., Feng S.S., Dong Y.X. (2018). Axial distribution characteristics of fragments of the warhead with a hollow core. Int. J. Impact Eng..

[11] Mott N.F. (1947). Fragmentation of shell cases. Proc. R. Soc. Lond. Ser. A.

[12] Grady D.E., Olsen M.L. (2003). Statistics and energy based theory of dynamic fragmentation. Int. J. Impact Eng..

[13] Grady D. (2006). Fragmentation of Rings and Shells.

[14] Taylor G.I. (1963). The fragmentation of tubular bombs. Advis. Counc. Sci. Res. Tech. Dev..

[15] Hoggatt C.R., Recht R.F. (1968). Fracture behavior of tubular bombs. J. Appl. Phys..

[16] Martineau R.L., Anderson C.A. (2000). Expansion of cylinder shells subjected to internal explosive detonations. Exp. Mech..

[17] Hu H.B., Tang T.G., Hu B.Y., Wang D.S. (2007). Longitudinal propagation of fracture surface in cylindrical metal shells under explosive loading. Shock Compress. Condens. Matter.

[18] Goto D.M., Becker R., Orzechowski T.J., Springer H.K., Sunwoo A.J., Syn C.K. (2008). Investigation of the fracture and fragmentation of explosively driven rings and cylinders. Int. J. Impact Eng..

[19] Tang T.G., Li Q.Z., Sun X.L., Sun Z.F., Jin S., Gu Y. (2006). Strain rate effects of expanding fracture of 45 steel cylinder shells driven by detonation. Explos. Shock Waves.

[20] Liu M.T., Ren G.W., Fan C., Tang T.G., Wang X.Y., Hu H.B. (2017). Experimental and Numerical Studies on the Expanding Fracture Behavior of an Explosively Driven 1045 Steel Cylinder. Int. J. Impact Eng..

[21] Avriel E., Lovinger Z., Nemirovsky R., Rittel D. (2018). Investigating the strength of materials at very high strain rates using electro-magnetically driven expanding cylinders. Mech. Mater..

[22] Singh M., Suneja H.R., Bola M.S., Prakash S. (2002). Dynamic tensile deformation and fracture of metal cylinders at high strain rates. Int. J. Impact Eng..

[23] Arnold W., Rottenkolber E. (2008). Fragment mass distribution of metal cased explosive charges. Int. J. Impact Eng..

[24] König P.J. (1987). A Correction for Ejection Angles of Fragments from Cylindrical Wareheads. Propellants Explos. Pyrotech..

[25] Wang L., Han F., Zhou Q. (2017). The projection angles of fragments from a cylindrical casing filled with charge initiated at one end. Int. J. Impact Eng..

[26] Taylor G. (1941). Analysis of the explosion of a long cylindrical bomb detonated at one end. Sci. Pap. GI Taylor..

[27] Johnson G.R., Cook W.H. A constitutive model and data for metals subjected to large strain, high strain rates and high temperatures. Proceedings of the 7th international symposium on ballistic.

[28] Li Y.X., Li Y., Yang M.S., Wei F.Z., Yuan Q.L., Cui F.K. (2015). Determination of 40Cr Johnson-Cook Dynamic Constitutive Equation for Cold roll-beating Forming Process. Chem. Eng. Trans..

[29] ANSYS AUTODYN. https://www.ansys.com/products/structures/ansys-autodyn.

[30] Kasvayee K.A., Ghassemali E., Salomonsson K., Sujakhu S., Castagne S., Jarfors A.E.W. (2017). Strain localization and crack formation effects on stress-strain response of ductile iron. Mater. Sci. Eng. A.

[31] Abuzaid W., Sehitoglu H., Lambros J. (2013). Plastic strain localization and fatigue micro-crack formation in Hastelloy X. Mater. Sci. Eng. A.

